# Are regulations addressing farm animal welfare issues during live transportation fit for purpose? A multi-country jurisdictional check

**DOI:** 10.1098/rsos.231072

**Published:** 2024-01-24

**Authors:** Eugénie Duval, Benjamin Lecorps, Marina A. G. von Keyserlingk

**Affiliations:** ^1^ Essex Law School, University of Essex, Colchester, UK; ^2^ Animal Welfare Program, Faculty of Land and Food Systems, The University of British Columbia, Vancouver, British Columbia, Canada; ^3^ Animal Welfare and Behaviour Group, School of Veterinary Science, University of Bristol, Bristol, UK

**Keywords:** animal transport, welfare, policies, law

## Abstract

Growing animal welfare concerns have pushed some jurisdictions to strengthen regulations addressing live farm animal transportation, but whether they provide satisfactory levels of protection for animals remains to be shown. Using the recent peer-reviewed literature, we identified four major risk factors associated with live animal transportation (fitness for transport, journey duration, climatic conditions and space allowances) and explored how regulations were structured to prevent animal welfare issues in five English-speaking Western jurisdictions (Australia, Canada, New Zealand, the EU and the USA). All legally binding federal regulations were systematically reviewed and compared. Whether these rules were fit for purpose was assessed using the relevant peer-reviewed scientific literature. Our findings indicate the majority of regulations in most jurisdictions are often insufficient or too vague to be deemed fit for purpose. All five jurisdictions fall short in guaranteeing adequate protection to livestock during transport. Using recent changes as well as future policy proposals under discussion, we identify future directions that could form the basis for regulatory changes that may significantly improve the welfare of farm animals during transportation.

## 1. Introduction

Agricultural practices are under increased scrutiny given their major contribution to climate change [1], biodiversity loss [2] and the increased awareness of animal welfare issues by the public [3]. The latter is one of the key barriers hindering the social sustainability of the livestock industries [4] with some food animal products or practices being phased out in some jurisdictions due to animal welfare concerns. For example, foie gras, which is produced by force-feeding ducks or geese, has been banned in several countries including Israel, Turkey, Argentina and most EU Member States due to animal cruelty concerns [5]. The European Union has also banned the practice of housing sows in gestation stalls for the duration of their pregnancy, with the exception of the first four weeks [6], for animal welfare reasons. The transport of live farm animals is another practice that affects most farm animals at some point during their lifetime and has attracted growing criticism due to animal welfare concerns.

Farm animals are typically subjected to transport at least once in their lifetime (e.g. from the farm to the abattoir) but in some animal industries, transport may also occur during other parts of the production cycle, frequently coinciding with a change in ownership. In North America, pigs are often transported as weaners from farms that specialize in producing piglets to farms that specialize in fattening; in some cases, the newly weaned pigs may be transported long distances across the country [7]. Animals are also often transported to auctions before being further transported to slaughter or fattening facilities (e.g. veal calves [8]). For broiler chickens, transportation will happen twice in their lives (i.e. as day-old chicks from the hatchery to the grower facility and as adults from the grower facility to the slaughterhouse [9]). Transportation is a stressful experience for animals (e.g. for cattle [8]); in most cases, animals are prevented from drinking, eating and resting during transport which can be very long. For example, in Canada, some animals (e.g. cattle) can be transported for 36 h without feed, water and rest. Live transportation is often associated with animals being exposed to additional stressors, such as comingling with unknown animals, human handling and extreme temperatures. Live transportation is therefore especially challenging for vulnerable animals (e.g. cull sows [9]; cull cows [10]); classes of animals that are usually not exempt from long journeys in some jurisdictions (e.g. the EU).

Most jurisdictions have put regulations in place to protect animals from harm. However, there is an increasing number of public reports citing catastrophic and systemic failures in protecting animals during live transportation. Challenges associated with live animal export to third countries (non-EU members) have been highlighted in the EU [11] and New Zealand banned the export of livestock by sea effective on 30 April 2023 following the sinking of a ship departing from New Zealand with 43 crew members and 6000 cattle on board [12]. These events strengthened views amongst the public that livestock are not effectively protected from harm during transport (see review [13]; Europe [14]; Australia [15]; Canada [16]). This view is also shared by some institutions; members of the EU Parliament have repeatedly called for improved enforcement of existing regulations and for new, more protective regulations during farm animal transport [17–19].

Global trade agreements between countries put additional pressure on jurisdictions to adopt regulations that are efficient and harmonized between jurisdictions. Trade restrictions based on animal welfare concerns have been implemented based on World Trade Organization rules [20,21]. Bilateral trade agreements in the future may involve more discussions on how the animals were cared for in the country of origin, including how they were transported and the degree to which the regulations protected the animals. In the context of the EU ‘Farm to Fork Strategy’, the introduction of ‘mirror clauses’ (i.e. clauses that would require imports from third countries to adhere to the same welfare standards required by the EU) in trade agreements with non-EU countries have been called for by some stakeholders and several EU ministers [22]. Such clauses could potentially improve the welfare of farm animals beyond the EU borders [23]. However, if global trade agreements are based on harmonized rules, these rules must first demonstrate that they are fit for purpose.

Here we aim to provide the first comprehensive multi-country jurisdictional scan—or fitness check—of live animal transportation regulations in five English-speaking Western jurisdictions (i.e. Australia, Canada, New Zealand, the EU and the USA). Fitness checks are defined by the European Commission ‘as a comprehensive policy evaluation to assess whether the regulatory framework for a policy sector is fit for purpose’ [24]. This type of scan can help jurisdictions draw conclusions on potential future regulatory changes. Our analysis is intended to enrich this process by evaluating five jurisdictions rather than one (e.g. [25]). This multi-jurisdiction approach provides a broader perspective on the different regulatory tools available to address similar issues.

We explored how these jurisdictions have approached the issues associated with live animal transportation with a focus on binding regulations. We did not explore non-binding recommendations and guidelines (also known as soft laws). In some circumstances, soft laws can be useful [26,27] notably to disseminate new rules more quickly between stakeholders (e.g. in Canada, Appendix L, ‘Should this pig be loaded?’ Decision Tree [28]), but they suffer important limitations, especially if they are not officially adopted as complementary to an already comprehensive set of binding rules (e.g. [29,30]). Hence, the first step is to document how different jurisdictions set up comprehensive binding regulations to protect animals during live transportation.

**Figure 1.**
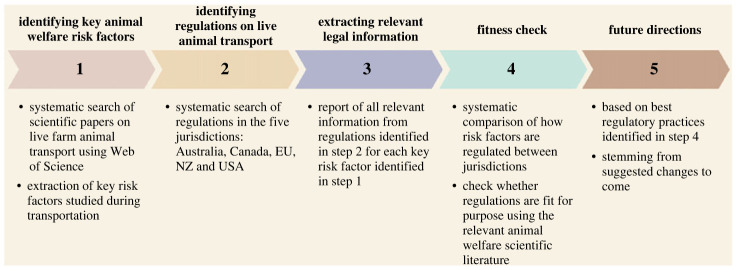
Methodological diagram representing the different steps of the analysis.

To assess how the five different jurisdictions address live farm animal transportation, we aimed to (i) identify major risk factors during transport using a systematic search of the relevant peer-reviewed scientific literature, (ii) identify and screen all relevant legal texts in the five different jurisdictions addressing each of the different risk factors identified previously, (iii) systematically check and compare how the five jurisdictions address risk factors in accordance with the scientific literature to highlight major gaps in current policies and areas where regulations appear fit for purpose and finally (iv) propose future policy directions inspired by the most comprehensive regulations identified in the comparative analysis and from plans highlighted by different countries for future regulations (figure 1).

## 2. Material and methods

### 2.1. Identification of main risk factors

To identify the main risk factors leading to animal welfare issues during live transportation of farm animals, we first conducted a rapid systematic search. Using Web of Science, we searched the peer-reviewed literature published between January 2021 and December 2022 using the following Boolean search terms in the Web of knowledge database: livestock OR calf OR calves OR cow* OR pig OR piglet OR chick* OR ‘laying hen’ OR lamb OR sheep OR sow OR goat* OR rabbit* OR cattle OR horse* OR turkey, AND lairage OR transport OR transportation OR ‘Live animal transport*’, AND ‘animal welfare’ OR ‘animal wellbeing’ OR ‘animal well-being’ OR stress. Our assumption was that the recent scientific literature would have largely focused its efforts on the most pressing issues.

We included papers published in peer-reviewed, refereed journals. We sorted articles related to ‘dairy and veterinary sciences' and excluded reviews and conference abstracts. This process resulted in 214 published papers. Titles and abstracts were then read and only papers focusing on live animal transportation welfare were included. To be included, papers had to explore risk factors for reduced farm animal welfare during transportation (studies looking at what happens before transportation or after it ends were not considered). We extracted the aim of each study to identify which key risk factors were investigated. Aspects related to the journey, such as length, breaks and distance covered, were grouped as ‘journey duration’. Aspects related to trailer type, ventilation, heat zones, temperature, humidity and CO_2_, season or time of the day were grouped as ‘climatic conditions’. Factors related to space allowances and density were grouped as ‘stocking density’. Pre-transport refers to studies looking at different animal management practices (e.g. fasting) before transportation, which can affect an animal's response to transportation. The systematic search was not intended to identify all factors affecting animal welfare during transport, but to highlight some of the key issues. We encourage future research to broaden the scope of their research to identify potential gaps in the literature.

### 2.2. Jurisdictions examined

This study is based on a comparative analysis of the rules that govern livestock transport in five Western English-speaking jurisdictions: Australia, Canada, New Zealand, the EU and the USA. Our analysis also covers the UK as farm animal transportation is still under the EU Transport Regulation. Billions of animals are transported each year in these jurisdictions (e.g. over 1.6 billion live animals were transported in the EU and beyond its borders in 2019 [31]; in Canada over 700 million animals are transported each year [32]). We focused on these five jurisdictions because they display comparable levels of development [33] and have some similarities in their farm animal industries. However, the same issues may not have been addressed similarly in the different jurisdictions given differing geographical constraints, legal frameworks or timelines in adopting transportation regulations. The comparison between these five jurisdictions not only covers different parts of the globe but also provides opportunities to understand the different ways in which countries regulate live animal transportation.

We focused our analysis on current mandatory rules and excluded ‘soft’ instruments such as non-binding recommendations and guidelines. We used the term ‘regulations’ throughout this paper, but in some jurisdictions, rules applying to the transport of farm animals are written in codes of practice but not included in legislations per se. We only included codes of practice for jurisdictions where they are binding legal instruments. A full list of law materials used for this paper can be found in electronic supplementary material, table S1.

We also limited our investigation to regulations adopted at the national or federal level. For example, in the USA, this meant that our investigation was mostly limited to the so-called federal ‘Twenty-Eight-Hour Law’ and in the EU, we used the Transport Regulation that is applied to each EU Member State. Lastly, in the case of Australia, Canada and New Zealand, we used national legislation or mandatory standards. However, there may be variations within a jurisdiction. For example, although we used the national standards for land transport as a basis of comparison in Australia, there are minor differences between states. Member states, provinces or territories may also adopt additional regulations, depending on the legal framework within each country.

We identified the main policy texts legislating live farm animal transportation in each jurisdiction. Each regulation/policy document was then screened for any relevant information regarding the four animal welfare risk factors (e.g. any mention of regulated journey duration of live farm animals). This allowed us to compare jurisdictions based on the type of regulations that have been implemented to protect farm animals during transport. Data were extracted from these regulations that related to the four key animal welfare topics. Data extraction included reviewing all relevant sections within the legal texts. If additional regulatory guidance existed to help stakeholders implement the legislation, this was also considered. For example, in Canada, the Interpretive Guidance Document helps stakeholders to interpret the regulations, especially when the wording is vague [34].

### 2.3. Fitness check

After summarizing and comparing regulations in the five jurisdictions, we reviewed the animal welfare scientific literature for each of the four risk factors to assess and compare whether the different jurisdictions adopted regulations that are fit for purpose. The legal information was checked against the available scientific literature, specifically looking for convergence (when a legal text applies current scientific consensus) and divergence (when a legal text does not apply current scientific consensus) on each topic. Lastly, using different sources such as the recent European Food Safety Authority (EFSA) reports [35–39] and the Inception Impact Assessment ‘Revision of the EU legislation on animal welfare’ [40], we considered both recent and proposed changes to the regulations. In the latter case, this refers to reviewed changes that have been announced but have not yet been translated into legislation or to different options that are being considered.

## 3. Results and discussion

Among the 214 papers published between January 2021 and December 2022 on the welfare of farm animals during transport, 58 (27%) met our inclusion criterion. Our results indicate that the most researched topics included studies that focused on climatic conditions (*n* = 28), journey duration (*n* = 28), stocking density (*n* = 14) and fitness for transport (*n* = 4). Other factors (e.g. motion stress, environmental enrichment or species transported) were explored in only one or two studies. Based on these findings we retained four main risk factors: climatic conditions, journey duration, stocking densities and fitness for transport. That said, we do recognize that other potential animal welfare risks including challenges associated with loading (e.g. handling; ramp design), mixing animals, pre-transport management (e.g. fasting) or driving conditions [41] are also important and warrant future work.

### 3.1. Fitness for transport

A comparative analysis of the regulations in the five jurisdictions can be found in electronic supplementary material, table S2. The transport of unfit animals has been cited as ‘the single most important [animal welfare] issue’ during transport [42] as it can impose undue suffering, particularly when the animal's condition deteriorates during transport [43]. Reasons contributing to poor fitness for transport may include young age, advanced pregnancy or the presence of one or more health conditions (e.g. in pigs [44]). In dairy cattle production systems, culling (removal from the herd) of sick (e.g. mastitis, metritis) or lame [45] cattle increases the risk that transportation can further compromise the animal, inducing additional pain and suffering and leading to a higher risk of mortality over long journeys [46]. Similar problems exist for cull sows [47]. Compromised animals may also become ‘downers’ during transport, a term commonly used to describe animals unable to stand (e.g. [48]).

The federal legislation in the USA, adopted in 1873, does not include regulations on fitness for domestic transport apart from a ban on the slaughter of non-ambulatory cattle [49]. The USA does, however, have some limited requirements on fitness for transport in regulations governing live animal exportation (electronic supplementary material, table S2). In contrast, the other four jurisdictions have all adopted more comprehensive regulations and generally prohibit the transportation of unfit animals. For example, in Canada, the revised animal transport regulations now state ‘no person shall load, confine or transport an animal that is unfit’ unless certain conditions (e.g. if the animal is to receive care) are met. However, there is some evidence that education about the regulatory specifications about fitness for transport is lagging for some key stakeholders (e.g. in Canada: Atlantic Canada, dairy farmers [50]; British Columbia, livestock hauliers and dairy farmers [51]; Ontario, dairy farmers [52,53]; Ontario, veterinarians [54]).

Regulations tend to prioritize aspects of what makes an animal ‘unfit’ and rarely include attributes of what makes an animal fit for transport [44], though Australia does include some elements defining an animal fit for transport. However, the list of ‘unfit’ signs ranges greatly across jurisdictions. For instance, EU regulations only include two clinical-related signs (i.e. animals ‘unable to move independently without pain or to walk unassisted’; animals presenting ‘a severe open wound, or prolapse’). In contrast, the newly revised Canadian legislation provides an extensive list (e.g. ‘non-ambulatory’; ‘in shock or dying’; ‘has a severe open wound or a severe laceration’). Australia, likely because this country has a long history of live transport by sea, appears to have higher expectations regarding the fitness for transport of exported live animals compared to inland transport and to other jurisdictions (see electronic supplementary material, table S2).

The legislative requirements in most jurisdictions are often written in ambiguous language. While conditions that make an animal unfit for transport are sometimes explicitly described, such as forbidding the transportation of animals that are in late pregnancy (e.g. last 10% of the gestation period, Canada and the EU), have recently given birth (e.g. during the preceding 48 h in Canada; during the preceding week in the EU) or if they are too young, often the language is either broad or subject to interpretation [55]. For example, an animal that is ‘extremely thin’ (Canada) or ‘unable to move independently without pain or to walk unassisted’ (EU) is subjective and thus open to interpretation by those deciding whether the animal is fit for transport or not.

In Denmark, approximately one-third of the 119 dairy farmers who responded to a questionnaire (2500 Danish dairy farmers were sent the survey) reported experiencing doubts about fitness for transport, especially around lameness [56]. Also in Denmark, 35% of cattle truck drivers surveyed (approx. 55% of all registered Danish livestock drivers) reported being regularly in doubt as to whether the animal was fit for transport when assessing fitness for transport [57] doubts that may come from a lack of training or knowledge about the regulations. For instance, despite 94% of Danish livestock drivers declaring that they were knowledgeable about the EU legislation on fitness for transport, only 52% of the participants were able to correctly answer two questions on the legislation [57]. In Canada, fever is one of the signs mentioned in the definition of unfit animals and animals in peak lactation are considered compromised; however, a recent survey of Ontario farmers (7.4% response rate) showed that lactation status and fever were considered by some farmers (i.e. 28 and 15%, respectively) as ‘unimportant or of little importance’ when assessing if a cow is fit before transport [52].

The line between a compromised animal (can be transported) and an unfit animal (cannot be transported, with exceptions) is often thin. In the EU, ‘slightly injured or ill’ animals may be considered fit for transport. However, the word ‘slightly’ is vague and is open to interpretation [58]. To our knowledge, Canadian lawmakers are unique in including a specific section entitled ‘Compromised animals’ that is accompanied by a list of clinical signs (e.g. an animal that ‘has acute frostbite’ or ‘is blind in both eyes') (see electronic supplementary material, table S2). Additional and specific conditions must be met before transporting these animals (e.g. animals must be transported to the nearest place, other than an assembly centre). Although little is known about whether these mitigation measures are effective in limiting the suffering of the animals during transport [43], adjusting transport rules for animals with disabilities or conditions likely to make them especially vulnerable is an improvement.

In some jurisdictions, veterinarians are asked to provide a certificate to attest if the animal is fit for transport. For example, in New Zealand, a lame animal must not be subjected to transport unless accompanied by a veterinary certificate that states otherwise. However, hundreds of unfit animals were transported in one EU Member State with veterinary certificates that failed to report health and welfare issues [59] so this approach should be viewed with caution. Challenges may come from the absence of clear guidance about fitness for transport, which may result in variation between veterinarians when assessing an animal's fitness for transport. When investigating differences in assessments of fitness for transport between Danish farmers, veterinarians and livestock drivers, authors reported that the level of agreement within and between each of these groups was at best ‘moderate’ [58]. A recent survey of veterinarians' practices and attitudes around cull cow management in the Canadian province of Ontario may also indicate a lack of education and training [54]. Although a large majority of the participants (i.e. 82.5%) reported being familiar with the new transport legislation adopted in February 2020, over a third of the respondents reported being interested in learning more about the regulations (i.e. 37%) and fitness for transport assessment.

Overall, with the exception of Canada, the other reviewed jurisdictions have limited regulations on fitness for transport. Most regulations provide some information on signs of what makes an animal unfit but continue to allow vulnerable animals (e.g. cull animals; ‘slightly injured or ill’ animals, EU) to be transported in the same way as fit animals.

### 3.2. Journey duration

A comparative analysis of the regulations in the five jurisdictions can be found in table 1 and electronic supplementary material, table S3. Journey duration is an important issue that can have a profound impact on animal welfare. Any journey, whether it is long or short, likely affects the welfare of animals (e.g. in pigs [60]), but long journeys can exacerbate the negative effects associated with transportation, such as food deprivation or exposure to extreme temperatures. It is well established that animals transported during long journeys are at greater risks for compromised welfare [41]. Increased travel duration has been associated with increased mortality in cattle [61] (for contrasting results, see [62]), calves [63], pigs [50,51]; especially when associated with elevated temperatures [64] (for contrasting results, see [65]; for a review, see [66]) and poultry [55–57,59,60]. An increase in stress biomarkers following transport has been reported, for example, in pigs [67], horses [68] and cattle [69]. Long journeys can also cause dehydration (e.g. in horses [70]) and increased body weight loss (e.g. in cattle [46]). Some animals, such as cull dairy cows, are more vulnerable to long transport [46] but may be subjected to long transit times in some jurisdictions (e.g. some cows are in some cases reported to be in transport for 7–10 days according to Canadian stakeholders [71]).

**Table 1.** Table summarizing specific regulations for journey durations by road for young calves in five different jurisdictions.

jurisdiction	age range for specific regulations (in days)	maximum duration/distance applied
Australia	≤5	6 h
Canada	≤8	12 h
EU	<10	100 km
	between 10 and 14	8 h
New Zealand	≤14	12 h
USA	—	—

Regulations either provide a maximum total duration (i.e. animals must arrive at their final destination within a certain time) or a maximum duration without feed, water and rest. In the latter case, animals can be transported indefinitely if some requirements, such as rest stops, are met. In four of the five jurisdictions examined (exception being the USA), maximum durations for the entire journey have been adopted, but only for some species or some animal categories. For example, in Canada, calves aged 8 days or less, and in New Zealand calves aged 14 days or less, can only be transported once for a maximum duration of 12 h (table 1). The changes in New Zealand were driven by public outcry after the release of animal cruelty footage on a dairy farm in 2015 [72,73]. Although an improvement, there are concerns that the 12 h maximum duration is still too long [72]. In Australia, the maximum duration of transport for calves aged 5 days or less when transported directly to a calf-rearing facility is 6 h. In the EU, maximum durations are provided for chicks (24 h) and poultry and rabbits (12 h) but only for journeys without food and water. In the EU, unless livestock are transported less than 100 km, there are requirements based on a minimum age (e.g. calves must be at least 10 days old for journeys less than 8 h; 14 days old for journeys over 8 h). Newborns are especially vulnerable to transport in part because they require more frequent meals [74]. A recent study on calves showed that both the age of the animal (younger animals being more vulnerable) and the length of the journey can exacerbate the negative effect of transportation [75].

Except for the specific cases outlined above, none of the jurisdictions have adopted a maximum total (or ‘absolute’) duration of transport before arriving at the final destination. Instead, guidance is often provided regarding the maximum intervals that animals can go without feed, water and/or rest (electronic supplementary material, table S3). However, the language can be interpreted in ways that allow animals to be transported for an unlimited time if some requirements are met (e.g. rest periods). Most jurisdictions have adopted regulations for a broad range of species. In contrast, New Zealand does not have regulations on transport duration, meaning there are currently no rules requiring livestock transporters to stop and provide water or feed along the journey for any livestock species except young calves. Sea transport is generally dealt with separately as journey durations do not apply to this form of transport, with the exception of Canada for ‘roll-on-roll-off vessels' (i.e. animals transported on ‘roll-on-roll-off vessels’ are not unloaded from the trucks).

For the purposes of this discussion, we assume that all journeys start when animals last had access to feed, water and rest; the definition used in Canada. For additional context, note that the EU uses the time when the first animal is loaded onto the vehicle as the journey's start and there is no specification in US regulations. In Australia, only a maximum time without access to water is mentioned in regulations. From the animals' perspective, the time since their last meal or drink is most relevant given the negative effects associated with food and water deprivation (e.g. [41,76]). These effects are also likely exacerbated when animals are fasted before transport, a common practice for pigs (e.g. [77]).

Jurisdictions differ greatly in terms of maximum intervals that an animal can be transported without access to feed, water and rest (table 1). Australia, Canada and the EU state that adult cattle, sheep and goats must not be transported longer than 48, 36 and 29 h, respectively. In the EU, the 29 h rule can only be used if the animals are provided a mandatory 1 h stop after 14 h, during which they must be rested, watered and (if necessary) fed. There is no mandatory unloading during this time.

In the EU, livestock transporters must follow specified maximum durations and unload animals so that they can be fed, watered and rested for a certain period of time. In contrast, in Australia, Canada and the USA, lawmakers give more leeway to transporters if trailers meet specific requirements (e.g. allowing animals to be fed, watered and rested onboard). In Australia, livestock does not have to be unloaded during the time of the break, but the vehicle must be stationary. A similar provision exists in the USA, suggesting that the animals should be unloaded ‘unless there is ample room in the car for all of the animals to lie down at the same time’. However, in the USA, journey breaks are not mandatory for animals if they can eat, drink and rest during transport. Canadian transporters also have three options regarding duration times when it comes to transporting livestock. First, they are exempt from rules on maximum journey durations without food, water and rest if the vehicle is fully equipped with drinkers, feeders, resting space and environmental control and recording systems (i.e. no mandatory stop). Second, if the vehicle is not fully equipped but meets some requirements, journey durations apply (i.e. the truck must stop) but animals may stay onboard for the time of the rest period. Third, animals may be unloaded in an approved lairage facility.

The animal welfare benefits of rest stops are unclear. Some have argued that a mandatory rest stop extends an already long journey duration [41,78–80] and that the benefits of providing a rest period for calves may be limited [81–83]. However, other work provides contrasting evidence. For example, cattle provided a rest period of 24 h after transportation for long periods experienced improved recovery [84,85]. Although some jurisdictions allow transporters to keep cattle loaded during the rest period when certain conditions are met, few studies have compared on-trailer versus off-trailer lairage [86]. While unloading may allow animals to access feed and water and eventually recover from the journey, it does come with additional stress associated with handling and potential comingling with unfamiliar animals in unfamiliar environments [87]. In contrast, not unloading the animals often prevents them from eating, drinking and resting; even if water is provided in the truck, animals may either be reluctant to drink while the vehicle is moving or be unable to access the drinker due to other animals blocking access [88]. The lack of clarity in the scientific literature on whether rest stops benefit animal welfare and how long animals need to recover is reflected by differences in the regulations (electronic supplementary material, table S3). In sheep, studies indicate that providing a longer rest stop may be preferable when compared to a short one [89,90]. Messori *et al*. [91] suggested that a 16 h rest may be enough to allow sheep to recover from the journey. These results raise questions regarding the adequacy of the short rest stop durations in place in Canada (8 h) and in the USA (5 h). Finally, the benefits of providing a short mid-journey break are also unclear. In an EU study involving cattle, providing a single 1 h on-trailer break following 14 h of transport (adhering to EU legislation) did not serve its purpose as many animals did not drink during the stop [84]. Overall, despite no clear evidence in support of having rest stops, or whether unloading benefits welfare, it does seem reasonable to ensure that all animals have access to key resources such as water after a certain time in transport, particularly in cases of elevated temperatures.

Some regulations may also be difficult to apply in practice. For example, in the EU, after 9 h of transport, unweaned animals must be provided a minimum rest stop of 1 h, be given liquid and if necessary, fed. However, regulations do not specifically state that the animals must be unloaded during that time, which means the journey may be up to 19 h long before unloading. Questions regarding this process have been raised. For instance, during a public hearing for the European Parliament Committee on animal transportation in 2021, arguments were made that the majority of 56 trucks that were observed were not equipped with an adequate drinking system for unweaned animals. When hauliers were asked why they do not feed animals, ‘the drivers [responded by asking] how they should do it. In fact, if you ever stand in front of a truck loaded with 220 calves on 3 decks, or 800 lambs on 4 decks, you understand why it's clearly impossible’ (I.B. 2021, personal communication) [92]. Problems may also include challenges associated with handling animals when unloaded to be fed, as this process is often done hurriedly due to time constraints (e.g. calves unloaded in France on their way from Ireland to The Netherlands). These findings and reports put into question whether the EU regulation of providing a mandatory short break during the journey is in fact effective in protecting animal welfare.

Driving times by the transporters are also regulated [25,93]. In the EU, during the 29 h of maximum travel time before a mandatory rest stop is needed for adult sheep, cattle and goats, the driver must stop four times (for 45 min; ‘drivers’ legislation). Long journeys may require several drivers being in the truck at the same time as each individual driver cannot exceed the maximum daily driving time of 9 h. In addition, the EU requires that there must be one 1 h break after 14 h of transport (‘animals’ legislation). The combined effect of these two pieces of legislation increases the time that animals spend in transport and the time they are left in a stationary vehicle, which can prolong exposure to extreme temperatures due to a lack of proper ventilation. Thodberg *et al*. [94] reported that the temperature inside Danish trucks transporting cull sows increased when the vehicle was stationary. Similar results were reported in cattle (Canada [95]), sheep (New Zealand [96]) and poultry (Canada [97]). This is more problematic for trailers that are only passively ventilated (i.e. through perforations in the walls), the norm for most livestock trailers used in North America [66,98].

Overall, all jurisdictions investigated in the current study allow animals to be transported for long periods (> to 8 h) without feed, water and rest. While the available evidence fails to provide information on how long is too long, there is agreement that deprivation of water, feed and rest during long journeys is detrimental to the welfare of the animals and should thus be reduced as much as possible. As mentioned above, Canadian and US regulations allow transporters to be exempt from maximum durations if their vehicles meet specific requirements despite that animals cannot properly drink, eat and rest onboard, especially when considering the typically low space allowances during transport (see below).

### 3.3. Climatic conditions

A comparative analysis of the regulations in the five jurisdictions can be found in electronic supplementary material, table S4. Although the importance of protecting animals from adverse weather conditions is recognized in most legislations, the majority have adopted vague rules on the topic, resulting in animals being exposed to extreme climatic conditions during transport (electronic supplementary material, table S4). For example, according to the federal standards for land transport in Australia, ‘a person in charge must take reasonable steps to minimize the impact of extreme weather conditions on the welfare of livestock during the transport process'. What ‘reasonable steps’ and ‘extreme weather’ mean is open to interpretation, although the standards provide some guidance on what is meant by ‘extremes of weather’ (i.e. ‘Temperature and climatic conditions (e.g. rain, hail, snow, wind, humidity and heat) that—individually or in combination—are likely to predispose livestock to heat or cold stress'). In particular, the absence of specific thresholds in most jurisdictions (Australia, Canada, New Zealand and the USA) makes it difficult to implement the requirement that animals must be protected from severe environmental conditions [48].

In contrast, the EU has adopted minimum and maximum temperatures inside the vehicles; ventilation systems ‘must be capable of maintaining a range of temperatures from 5°C to 30°C within the means of transport, for all animals, with a ±5°C tolerance’. Although this is more ambitious than other jurisdictions, it is far from perfect. For instance, these specific thresholds only apply to journeys longer than 8 h by road. Although the EU text states that this applies to ‘all animals’, the placement of this article in a chapter specific to long journeys for horses, cattle, sheep, goats and swine makes it unclear whether other animals, such as rabbits and poultry (e.g. for rabbits [99]; for poultry [100]), are also included despite evidence that they too can experience cold and heat stress during transport (defined as the situations in which ‘the animal experiences stress and/or negative affective states such as discomfort and/or distress when exposed to low (or high) effective temperature’ [101]). There is also no guidance regarding age, injuries or sickness despite these factors having a profound effect on how an animal is impacted when exposed to extreme temperatures [41]. There are also important species-specific differences in thermal comfort zones. For example, studies have found that the upper threshold of the thermal comfort zone is 20°C for sows and 25°C for sheep [36,101]. However, these temperatures are only estimates as most studies on this topic have not been done under realistic transport conditions. Pigs, poultry and rabbits are all highly sensitive to the effects of heat stress [66] while sheep appear to be less vulnerable than cattle [102]. Breed differences exist (e.g. in cattle [62]) and the production stage may also play a role (e.g. lactating versus non-lactating [103]).

As stated by Mitchell & Kettlewell [104], ‘It is not certain that the thermal limits prescribed in such legislation are entirely appropriate for all livestock’. The stated thresholds in the EU allow animals to be transported when temperatures are outside the thermal comfort zone of most species, and for extended periods of time. Several studies highlighted increased mortality rates with increased temperatures [105–108]. According to the EU legislation, pigs can be transported during long journeys at temperatures as low as 0°C and as high as 35°C; extremes well outside of animals' thermal comfort zone. A Danish study reported that temperatures inside the vehicles, especially during summer and autumn, were outside of the thermal comfort zone of sows [94] despite only one of the 39 journeys taking place when outside temperatures were above 25°C. In addition, the comfort zone of sows was established decades ago and may not be relevant to the modern breed given changes in genetics [94,109]. Recent studies reported that the upper threshold of the thermal comfort zone of sows may be lower than 20°C (e.g. late-gestation sows [109]; lactating sows [110]).

Lastly, the current regulations only refer to temperatures and do not mention other factors, such as air humidity, despite the importance of this factor on the temperature felt by the animals. Ambient temperature (outside and inside the vehicle) and relative humidity should be considered, given that the latter can clearly exacerbate the effects of heat stress [111]. Scientific evidence shows that with a 30°C limit (dry bulb temperature), the felt temperature ranges from 29 to 44°C with increasing humidity [112]. The temperature–humidity threshold for dairy cow heat stress is often set at 72 (albeit a conservative threshold), which can be reached at temperatures as low as 22°C when humidity is high [113]. However, in the EU, it is legal to transport cows when the dry bulb temperature is above 30°C (and up to 35°C, given the 5°C tolerance). The EFSA panel recently suggested other methods of calculation for heat stress that consider additional environmental factors [36].

Although limited, the EU legislation still provides a greater degree of protection compared to other jurisdictions that chose not to adopt specific thresholds. González *et al*. [48] reported that cattle transported to or from the Canadian province of Alberta were sometimes exposed to temperatures as low as −42°C and as high as 45°C. In Australia, several studies reported that cattle and sheep are especially at risk for heat stress when exported by sea to the Middle East during the Northern Hemisphere summer [114,115]. Following the airing of undercover videos showing deadly conditions during sea transport, an independent review commissioned by the Australian Government [116] led to the adoption of a ban of sheep exports to the Middle East between June and September in 2020 (a temporary ban was adopted in 2019 [117]). However, this ban has been partially lifted as sheep can now be exported to or via the Red Sea during the first two weeks of June [118]. Similarly, cattle cannot be transported south of latitude 26° to the Middle East during the Northern Hemisphere summer months, a limitation that existed before the adoption of temporary bans for sheep [102]. However, caveats remain that could still make it possible to export cattle during that time such as if the heat stress risk is deemed ‘manageable’ (i.e. less than 2% risk of a 5% mortality).

Lastly, most jurisdictions require adequate ventilation to limit thermal stress. However, in the case of road transport for example, regulations remain vague regarding the type of ventilation needed inside vehicles (i.e. passive/natural ventilation; or active/mechanical/forced ventilation). In Australia, New Zealand and the USA, although recommended, forced-ventilation systems are not mandatory for road transport. For this type of transport, stricter rules exist in the EU and in Canada but only in a limited number of cases and species (in the EU: for long journeys by road for horses, cattle, sheep, goats and pigs; in Canada: for journeys exempted on meeting the maximum intervals). Both jurisdictions require mechanical ventilation systems that must be maintained, as well as systems to monitor and record temperatures (and humidity, in Canada) inside vehicles. A warning system must be installed to alert the driver when temperatures reach a set temperature. Lastly, adequate ventilation must be provided ‘at all times’ according to Canadian legislation, which upon review also appears to include stationary periods, something that is also clearly specified in the EU Regulation. Mechanical ventilation, if adequately provided, may aid in limiting temperatures inside vehicles [104,119]. However, mechanical ventilation is not a panacea as it can fail to decrease the temperature of the animals inside the truck (pigs [120]).

Overall, these results raise questions regarding the adequacy of the regulations in the five jurisdictions. While most jurisdictions have only vague regulations on ventilation, EU regulations are more specific but do not reflect the latest scientific evidence.

### 3.4. Space allowances

A comparative analysis of the regulations in the five jurisdictions can be found in electronic supplementary material, table S5. Stocking density can affect the welfare of animals during transport [88]. However, optimal space allowances are not easily determined, as both too little or too much space per animal can have negative impacts (e.g. cattle [121]). For example, some have discussed whether cattle benefit from lower space allowances in some situations (e.g. poor driving conditions) to maintain their balance [122]. However, two studies also suggest that the lower the space allowance the greater the risk of stress and injuries [123,124].

The current regulations across all jurisdictions on stocking density mention that animals should be provided with enough space (electronic supplementary material, table S5). For example, New Zealand requirements state that ‘stocking density must be sufficient to allow animals to adopt a natural posture during the journey without injuring their heads or backs if they stand, and to allow animals to rest, if this is necessary during the journey’. In Canada, there is also explicit language stating that overcrowding the animals is forbidden, making specific reference to the animals' position within the truck (e.g. ‘the animal cannot maintain its preferred position or adjust its body position in order to protect itself from injuries or avoid being crushed or trampled’). In Australia (land transport) and the USA, specific space allowances are not written in laws (or in national standards in Australia), but are written in codes of practice or guidelines as non-binding recommendations. Minimum space allowances exist in Australia as binding regulations but only for export by sea or by air and for a limited number of species. In contrast, the EU has provided transporters with specific and mandatory minimum space allowances for different species depending on the type of transport (i.e. rail, road, air, sea) and the animals' weight. With the exception of the Australian standards for export, the EU minimum space allowances are either equivalent or have higher expectations than the recommendations and guidelines on space allowances in Australia (land transport) and the USA.

The EU regulations, although strict compared to the other jurisdictions, lack precision and, given that they were adopted in 2005, fail to reflect the latest scientific evidence. Although providing adequate space above the animal within the truck may be important, no jurisdiction provides specific height requirements (except the EU for horses), potentially due to the lack of scientific literature on this topic [125].

In terms of minimum floor space allowances for animals transported by road transport, recommendations are almost always based on weight and not body size, which can vary greatly [126]. To that end, the use of allometric equations for cattle, sheep and pigs ‘to estimate the volume of space an animal occupies as a function of its mass’ [127–129] may help. In the case of horses, stocking density (i.e. m^2^ kg^−1^) may be a more appropriate measure than the current reference to space allowance (i.e. m^2^ per animal), especially if their weights and body conditions differ [127] (for untamed ponies, see [130]).

Absolute space allowances may also fail to efficiently protect animals during transport. Although cattle prefer to stand during transport [131,132], lying down may be necessary during a long journey [132]. However, the lower limit of the EU space allowance fails to provide sufficient lying space and for movement between lying and standing and vice versa [127]. For instance, heavy cattle (i.e. 550–700 kg) need more than 3 m^2^ to move between lying and standing, which falls outside of the range stated by the EU Regulation for minimum space allowance (i.e. between 1.30 and 1.60 m^2^ [125]). Based on a comparison between the minimum space allowances recommended for cattle to lie down and the lower limit of the minimum space allowance of the Regulation, the lower range of the minimum space appears to consistently fall under the minimum spaces recommended by the available scientific literature for cattle to lie down [125,127]. Recent reports from animal advocacy groups have used this argument to highlight animal welfare concerns associated with transport in crowded vehicles, even if the transporter is in compliance with the legal space allowance (e.g. [133]).

Similarly, minimum space allowances specified by the EU Regulation are insufficient to allow sheep to adopt their preferred positions (see [128]). This was also highlighted as ‘unacceptable’ by the EFSA Panel on Animal Health and Welfare [127]. As the Regulation only specifies a minimum space for lambs above 26 kg (i.e. 0.20–0.30 m^2^), young lambs < 26 kg are often provided less than 0.20 m^2^ per animal. Menchetti *et al*. [134] report that 0.27 m^2^ is required to transport smaller lambs, suggesting that the current recommendations are insufficient to protect their welfare during transport.

Similar findings of insufficient space were also found for pigs [135], especially at high ambient temperatures and when pigs need to access water inside the truck [111]. As noted by Arndt *et al*. [135], ‘the minimal floor area offered on animal transportation vehicles, according to European legislation, is not sufficient to grant finishing pigs of modern genetic origin enough static space in the fully recumbent body position’. Another issue is the lack of specific densities for pigs that are less than 100 kg as the Regulation does not specify space requirements for smaller pigs. According to the EU Regulation, ‘the loading density for pigs of around 100 kg should not exceed 235 kg per m^2^’. However, this was recently criticized by Bracke *et al*. [111] as ‘this loading density is obviously wrong for the smaller weight ranges. You cannot physically keep 8 pigs of 30 kg each on one m^2^, without stacking them on top of each other’. Finally, there are also no recommendations regarding the minimum space allowances for rabbits during transport; some also argue that the prescribed weight ranges are too broad [136].

Overall, regulations remain vague on space allowances during transport and although the EU stands out from the other four jurisdictions, current space allowances allowed in the legislation are not in line with the latest scientific evidence.

### 3.5. Future directions/considerations

**Figure 2.**
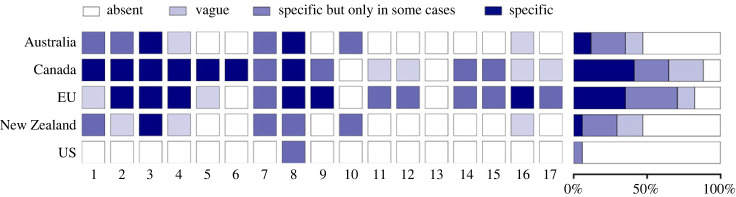
Mapping of the regulatory elements present in the federal regulations of the five jurisdictions for domestic journeys. Coloured segments indicate whether the issue is being regulated with different levels of regulatory efforts (absent, not regulated; vague, regulated using vague statements; specific but only in some cases, regulations apply only to some species or in some conditions; specific, precise regulations apply to all animals). Animal welfare regulations include: 1. Set a list of health signs defining unfit animals, 2. Considerations for pregnant animals, 3. Transport ban for unfit animals, 4. Transport ban without exceptions for unfit animals (unless required for veterinary care), 5. Set a list of signs for compromised animals, 6. Adapted conditions of transport for compromised animals, 7. Maximum journey durations (absolute/for the entire trip), 8. Maximum intervals without food, water and rest, 9. Rest stop with mandatory unloading, 10. Export ban, 11. Minimum temperatures, 12. Maximum temperatures, 13. Adopt temperature/humidity indexes, 14. Mandatory mechanical or forced-ventilation systems, 15. Mandatory monitoring, recording and alarm system, 16. Minimum space allowances, 17. Height requirements. The sum of all 17 factors is also provided in the form of a ‘heat map’ (right panel) to highlight how many of these 17 welfare indicators were regulated and to what extent in each jurisdiction (0% indicating none were addressed and 100% indicating all were addressed and colours refers to how precise regulations are).

To our knowledge, this paper is the first to provide an up-to-date evaluation and comparison of the regulations on live farm animal transport in several jurisdictions, highlighting similarities and differences but also trends for improvement and remaining gaps. Although variations between jurisdictions exist, our results show that all regulations, including the most recently revised ones, may not guarantee adequate protection to animals during transport. While some jurisdictions have made substantive advances on some issues, others do not address some issues, use vague language or do not reflect the latest scientific evidence. This comparative fitness check also highlighted areas where some jurisdictions provide clearer guidance than others (figure 2). Using these examples and new changes to come announced in several countries, we drew key future directions for regulatory changes.

#### 3.5.1. Unfit animals

Not all jurisdictions explicitly ban the transport of unfit animals nor provide clear definitions of unfit animals. Clear definitions of what makes an animal unfit (exemplified by the recent Canadian regulations) are needed to help decision-making of whether the animal is fit for transport. One way forward could be to establish a comprehensive list of clinical signs for each species. Unfortunately, the EU Commission proposal for a new Transport Regulation released in December 2023 [137] did not include such a list. It might also be useful to make additional materials including recommendations and decision trees that can help stakeholders make appropriate decisions about fitness for transport [43]. Some of the animal-based measures identified in the EFSA scientific opinion (e.g. lameness score, wounds, abscess, body condition score) could serve as a basis for future regulatory improvements [35–39]. We do, however, acknowledge that this is challenging as there is also a general lack of scientific agreement (EFSA Panel on Animal Health and Welfare [35–38,101]). Even with clearer legislations, assessing fitness for transport will likely remain a subjective task.

#### 3.5.2. Compromised and vulnerable animals

‘Compromised’ and ‘vulnerable’ animals are more likely to experience negative effects associated with transportation. Again, drawing from the Canadian example, a first step could be to clearly identify signs (e.g. young, pregnant, lactating and end-of-career animals) that an animal is unfit for transport, ‘compromised’ or ‘vulnerable’. Vulnerable animals require additional protection during transport such as ‘increased contingency planning, reducing journey duration, adjusting ventilation, increasing bedding, avoiding extreme weather conditions, avoiding loading via steep ramps, loading last and unloading first, providing space to lie down, increasing monitoring frequency, providing feed, water and rest more frequently and use of analgesics or other applicable medication’ [35]. The adoption of a maximum duration for the entire trip (rather than a maximum duration without rest) would limit the time animals are exposed to the negative effects associated with transport and prevent unnecessary loadings and unloadings. Banning long journeys (i.e. over 8 h) for ‘unweaned and other vulnerable animals' is specifically mentioned by the European Commission in its impact assessment for the future revision of the legislation [40]. Such a ban no longer appears in the recent proposal of the EU Commission. According to the draft proposal, calves would now have to be older than five weeks and lambs older than one week (instead of 10 days for calves and one week for lambs currently) before they could be transported unless they are transported for a journey less than 100 km [137]. However, unweaned animals could still be transported for a long time if the truck is equipped with a feeding system (i.e. 9 h followed by a rest period of 1 h without unloading followed by another 9 h). In addition, the proposal explicitly indicates that when the journey includes a transport by sea, the time spent at sea does not count as part of the journey time [137]. Finally, ‘pregnant females for whom 80% or more [instead of 90% in the current legislation] of the expected gestation period has already passed’ would now be considered as unfit for transport [137].

#### 3.5.3. Maximum durations

The EU 2005 Regulation states that ‘for reasons of animal welfare the transport of animals over long journeys, including animals for slaughter, should be limited as far as possible’. This language is, however, insufficient in limiting journey durations. In fact, between 2005 and 2015, the number of long journeys increased [138]. Reduced journey times are currently under consideration in the EU [113,115] (see also EFSA recommendations [35–39]) where some Member States already limit journey durations taking place within their borders to 8 h (e.g. cull sows in Denmark and Sweden [47,139]). For species other than poultry and rabbits for which EFSA recommends a maximum journey duration of 12 h, the EFSA report only highlights the importance of keeping journey durations to a minimum, without providing specific limits. The EU Transport Regulation draft released by the Commission in December 2023 includes reduced maximum journey durations. The draft proposes a maximum of 9 h for animals transported to slaughter, unless ‘no slaughterhouses adapted for slaughter of the species and categories of animals…can be reached within a short journey’ [137]. Regarding long journeys for a purpose other than slaughter, transportation would be allowed for a maximum of 21 h repeatable once. This would also include rest periods of 1 h after 10 h and a 24 h rest after 21 h during which animals would need to be unloaded. These changes would not apply to domestic birds and rabbits. Following recent public consultations in the UK [140], the UK and Welsh governments committed to introduce ‘absolute’ maximum journey times, including 4 h for broiler chickens, 9 h for calves up to nine months old, 12 h for horses and newly weaned pigs, 18 h for pigs, 21 h for cattle, sheep and all other animals and 24 h for recently hatched chicks. Whether this will translate into laws, and if so when, remains unknown. Revisiting the absolute maximum durations for the entire journey should be considered given that in some situations it appears that not all animals are able to properly eat, drink and rest during transport, a situation that compromises the welfare of some individuals being transported.

#### 3.5.4. Live animal export

The issue of live animal transportation is further complicated when animals are sold live and exported to another country, as the country of origin loses its capacity to ensure animal welfare beyond its borders. To be exported outside the EU's borders, the journey must, in theory, comply with EU regulations even when outside EU's borders [141]. However, as noted by the European Parliament, ‘there is no control system currently in place for transport to third countries, leading to situations where animal exports to third countries often do not respect Regulation (EC) No 1/2005 and are often in violation of the Court of Justice ruling C-424/13 on this matter’ [19]. The New Zealand government was the first to announce a ban on live farm animal exports (i.e. sheep, cattle, deer and goats) for slaughter by sea as of April 2023. In December 2023, a Bill including a ban on the export of livestock for slaughter and fattening from Great Britain (i.e. England, Scotland and Wales, but not Northern Ireland) was introduced in Parliament [142]. The export of livestock for other purposes, such as breeding, competitions and races, which account for the vast majority of farm animals exported by the UK, would still be permitted [142]. Additionally, the ban would apply to cattle, horses, sheep, goats, pigs and wild boar but not to other species such as poultry [142]. In 2020, more than 12 million poultry were exported from Great Britain to the EU [143]. In the EU, Germany has recently restricted the export of cattle, sheep and goats to third countries (i.e. non-EU countries). Veterinary certificates which are required for export will no longer be issued for fattening, slaughtering and breeding as of July 2023; a step that effectively prevents the animal from being exported. However, as noted by the German Federal Ministry of Food and Agriculture, these changes do not prevent German transporters from moving animals to another country and exporting them from there [144]. To that end, five EU Member States (i.e. Belgium, Denmark, Germany, The Netherlands and Sweden) called for an EU ban ‘on certain long journey exports of live animals to third countries by road and sea’ [145]. Banning the export of ‘certain categories of animals' is one of the options mentioned by the EU Commission in its impact assessment for the revision of the Transport Regulation [40]. This, however, is no longer proposed by the EU Commission as the draft revised legislation does not include a ban on live animal export [137]. Despite the adoption of several reforms aimed at improving animal welfare during export (see [114]), it remains to be seen what Australia does given that a large proportion of live sheep are exported annually. Although the current Australian government is committed to ban live sheep export (but not cattle), to our knowledge no timeline accompanied this announcement [146]. If confirmed, the current trend towards a ban on live animal export by some jurisdictions may lead other countries to adopt similar legislation. Although this may not necessarily result in other countries adopting similar legislation [147], the increasing number of international bilateral trade agreements that include animal welfare considerations (e.g. the EU-Chile Free Trade Agreement, described by von Keyserlingk & Hötzel [21]) may push countries to seek greater alignment in their respective regulations in order to remain competitive. This could include, among other considerations, the question of whether live animal export is allowed.

#### 3.5.5. Climatic conditions

Potential pathways moving forward for all jurisdictions would be to provide clear species/age/condition-specific thresholds for the temperature–humidity index inside vehicles (and not the outside or bulb temperature). There are some indications that lower climatic thresholds may be adopted in the UK [140]. In the EU, the draft revision released by the EU Commission includes stricter regulations; however, it would not prevent animals being transported in extreme temperatures (i.e. when the temperature forecast indicates certain temperatures, animals could only be transported for a limited amount of time and/or during a specific period of the day and/or with an increased space allowance [137]). According to EFSA, animals should be transported at temperatures within their thermal comfort zone and the temperature inside vehicles should not exceed their upper critical temperature (UCT) (i.e. the UCT is 25°C, for cattle, pigs and horses [35,36,38]. The adoption of adapted climatic thresholds would require trucks to be equipped with climate-control and mechanical ventilation systems. Some trucks already allow animals to be transported under temperature-controlled conditions (e.g. with air conditioning). Equipping vehicles with monitoring and recording systems for temperature and humidity (as is now the case for some journeys in Canada) could also allow the authorities to check the temperatures and humidity inside vehicles at any time, including after the end of the journey (for an example of a climate monitoring equipment allowing one to automatically calculate the temperature–humidity index, see [148]).

#### 3.5.6. Space allowances

Space allowances should allow animals to adopt their preferred postures but also to rest and access drinkers inside vehicles (assuming that they have previous experience with the type of drinker, which is uncertain). One way forward would be for all jurisdictions to begin by establishing species/age/condition-specific minimum horizontal (i.e. floor space) and vertical (i.e. deck height) space allowances as a binding requirement in law. In the EU, the proposal released in December 2023 includes updated space allowances [137]. In the UK, changes are limited to new headroom allowances. Again, it remains unclear whether these announcements will be translated into laws.

#### 3.5.7. Enforcement

Beyond the adoption of new rules, it is crucial that regulations are enforced. All jurisdictions studied share, to some extent, issues in enforcing current transport regulations and more generally animal welfare legislations (see examples: Canada [149]; Australia [117]; New Zealand [150]; EU [25]). This can cause important discrepancies between what regulations state and what animals experience. In the USA, the Twenty-Eight-Hour Law appears either rarely or never enforced [46,145]. Non-compliances are regularly reported by animal welfare organizations and in some cases by scientists (e.g. in the EU [151,152]; in Canada [68]). Critics within the European Parliament have urged Member States to do a better job in enforcing the regulations [17–19]. Ways to improve compliance and enforcement may include increased and improved inspections [25,152], increased penalties but also economic incentives [153], as well as trainings and educational tools [57]. To ensure that regulations are complied with, regular comprehensive mandatory trainings should be adopted. Improving drivers' working conditions may also be an important factor to improve animal welfare during transport as poor working conditions are likely to negatively affect the welfare of the animals [154,155].

If implemented across jurisdictions, these policy changes could lead to substantive improvements in the welfare of transported animals. However, some of these may require profound transformational changes in our food production systems in line with the ‘Farm to Fork’ ambition of the EU aiming to promote a more sustainable food production system. Whenever possible, the transportation of meat should be preferred over transportation of live animals (e.g. [17,25,156,157]) and alternatives to live animal transport must be further explored. For example, on-farm slaughter may mitigate some of the issues associated with transportation, especially of vulnerable animals [158,159].

Finally, in some areas, the scientific literature is unclear. As noted by Herskin & Duffield [160], ‘scientific focus on animal transport is relatively new’; thus, there are numerous gaps within this body of research that require attention. For instance, there is limited information regarding deck height and varying results regarding the benefits of unloading animals during a rest stop [35,36,38,39,128]. In addition, the development of measures that better reflect the animals' perspective (see [161]) may help determine the appropriate transportation conditions for different animal species. Based on precautionary reasoning about animal sentience by Birch [162], we suggest that where doubts exist or if there is a lack of scientific evidence, lawmakers should strive to adopt solutions that are most likely to protect the animals.

## Data Availability

The data are provided in electronic supplementary material [163].

## References

[1] Clark MA, Domingo NGG, Colgan K, Thakrar SK, Tilman D, Lynch J, Azevedo IL, Hill JD. 2020 Global food system emissions could preclude achieving the 1.5° and 2°C climate change targets. Science **370**, 705-708. (10.1126/science.aba7357) 33154139

[2] Rigal S et al. 2023 Farmland practices are driving bird population decline across Europe. Proc. Natl Acad. Sci. USA **120**, e2216573120. (10.1073/pnas.2216573120) 37186854 PMC10214186

[3] Alonso ME, Lomillos JM. 2020 Consumers' concerns and perceptions of farm animal welfare. Animals **10**, 1-13. (10.3390/ani10030385) PMC7143148 32120935

[4] Sinclair M et al. 2022 International perceptions of animals and the importance of their welfare. Front. Anim. Sci. **3**, 97. (10.3389/fanim.2022.960379)

[5] Rochlitz I, Broom D. 2017 The welfare of ducks during foie gras production. Anim. Welf. **26**, 135-149. (10.7120/09627286.26.2.135)

[6] Council Directive 2008/120/EC of 18 December 2008 laying down minimum standards for the protection of pigs (Codified version), OJ L 47, 2009, pp. 5–13.

[7] Engebretson M. 2008 North America. In Long distance transport and welfare of farm animals, pp. 218-260. Wallingford, UK: CABI.

[8] Webb LE, Verwer C, Bokkers EAM. 2023 The future of surplus dairy calves—an animal welfare perspective. Front. Anim. Sci. **4**, 1228770. (10.3389/fanim.2023.1228770)

[9] Tuyttens FAM, Jacobs L. 2020 Improving welfare in catching and transport of chickens. In Understanding the behaviour and improving the welfare of chickens (ed. C Nicol), pp. 417-459. London, UK: Burleigh Dodds Science Publishing.

[10] Knowles TG. 1999 A review of the road transport of cattle. Vet. Rec. **144**, 197-201. (10.1136/vr.144.8.197) 10097341

[11] Marahrens M, Kernberger-Fischer I. 2021 Research for ANIT Committee—the practices of animal welfare during transport in third countries: an overview.

[12] McCurry J, Visontay E. 2020 Cargo ship with 43 crew and nearly 6000 cattle sank off Japan, survivor says. *The Guardian*. See https://www.theguardian.com/world/2020/sep/03/typhoon-maysak-ship-with-43-crew-and-nearly-6000-cattle-missing-off-japan (accessed 2 July 2023).

[13] Clark B, Stewart GB, Panzone LA, Frewer IKLJ. 2016 A systematic review of public attitudes, perceptions and behaviours towards production diseases associated with farm animal welfare. J. Agric. Environ. Ethics **29**, 455-478. (10.1007/s10806-016-9615-x)

[14] Vanhonacker F, Van Poucke E, Tuyttens F, Verbeke W. 2010 Citizens’ views on farm animal welfare and related information provision: exploratory insights from Flanders, Belgium. J. Agric. Environ. Ethics **23**, 551-569. (10.1007/s10806-010-9235-9)

[15] Buddle EA, Bray HJ, Ankeny RA. 2018 ‘I feel sorry for them’: Australian meat consumers’ cattle transportation. Animals **8**, 1-13. (10.3390/ani8100171) PMC6211102 30282909

[16] Spooner JM, Schuppli CA, Fraser D. 2014 Attitudes of Canadian citizens toward farm animal welfare : a qualitative study. Livest. Sci. **163**, 150-158. (10.1016/j.livsci.2014.02.011)

[17] 2019 European Parliament resolution of 14 February 2019 on the implementation of Council Regulation (EC) No 1/2005 on the protection of animals during transport within and outside the EU (2018/2110(INI)).

[18] 2020 European Parliament decision of 19 June 2020 on setting up a committee of inquiry to investigate alleged contraventions and maladministration in the application of Union law in relation to the protection of animals during transport within and outside the Union, and defining its responsibilities, numerical strength and term of office (2020/2690(RSO)).

[19] 2022 European Parliament recommendation of 20 January 2022 to the Council and the Commission following the investigation of alleged contraventions and maladministration in the application of Union law in relation to the protection of animals during transport within and outside the Union (2021/2736(RSP)).

[20] Howse R, Langille J, Sykes K. 2015 Pluralism in practice: moral legislation and the law of the WTO after seal products. Geo. Wash. Intl Law Rev. **48**, 81-150.

[21] Von Keyserlingk MAG, Hötzel MJ. 2015 The ticking clock: addressing farm animal welfare in emerging countries. J. Agric. Environ. Ethics **28**, 179-195. (10.1007/s10806-014-9518-7)

[22] Times TB. 2023 Animal welfare: the case for mirror clauses in trade agreements. See https://www.brusselstimes.com/212560/animal-welfare-the-case-for-mirror-clauses-in-trade-agreements (accessed 9 June 2023).

[23] Di Concetto A. In press. The double-edged sword: international law and its effects on EU farm animal welfare legislation. *Global J. Animal Law*. See https://ojs.abo.fi/ojs/index.php/gjal/article/view/1756 (accessed 2 July 2023).

[24] OECD. 2021 Competitiveness and private sector development competitiveness in south east Europe 2021: a policy outlook. Paris, France: OECD Publishing.

[25] Bachelard N. 2022 Animal transport as regulated in Europe: a work in progress as viewed by an NGO. Anim. Front. **12**, 16-24. (10.1093/af/vfac010) 35311188 PMC8929986

[26] Fraser D. 2015 Turning science into policy: the case of farm animal welfare in Canada. Anim. Front. **5**, 23-27. (10.2527/af.2015-0027)

[27] Broom DM. 2002 Does present legislation help animal welfare? Aus dem Institut für Tierzucht Mariensee **90**, 63-69.

[28] NFACC. 2014 Code of practice.

[29] Coglianese C. 2020 Environmental soft law as a governance strategy. Jurimetrics. Faculty Scholarship at Penn Carey Law **61**, 19.

[30] Koutalakis C, Buzogany A, Börzel TA. 2010 When soft regulation is not enough: the integrated pollution prevention and control directive of the European Union. Regul. Govern. **4**, 329-344. (10.1111/j.1748-5991.2010.01084.x)

[31] European Parliamentary Research Service. 2021 Protection of animals during transport: data on live animal transport.

[32] Government of Canada PW and GSC. 2016 Canada Gazette—regulations amending the health of animals regulations. See https://gazette.gc.ca/rp-pr/p1/2016/2016-12-03/html/reg2-eng.html (accessed 3 June 2023).

[33] United Nations Development Programme. 2022 The 2021/2022 human development report.

[34] Government of Canada CFIA. 2020 Health of animals regulations. Part XII. Transport of animals—regulatory amendment interpretive guidance for regulated parties. See https://inspection.canada.ca/animal-health/terrestrial-animals/humane-transport/health-of-animals-regulations-part-xii/eng/1582126008181/1582126616914 (accessed 16 February 2023).

[35] EFSA Panel on Animal Health and Welfare (AHAW) et al. 2022 Welfare of cattle during transport. EFSA J. **20**, e07442. (10.2903/j.efsa.2022.7442) 36092766 PMC9449995

[36] EFSA Panel on Animal Health and Welfare (AHAW) et al. 2022 Welfare of pigs during transport. EFSA J. **20**, e07445. (10.2903/j.efsa.2022.7445) 36092763 PMC9449989

[37] EFSA Panel on Animal Health and Welfare (AHAW) et al. 2022 Welfare of domestic birds and rabbits transported in containers. EFSA J. **20**, e07441. (10.2903/j.efsa.2022.7441) 36092767 PMC9449994

[38] EFSA Panel on Animal Health and Welfare (AHAW) et al. 2022 Welfare of Equidae during transport. EFSA J. **20**, e07444. (10.2903/j.efsa.2022.7444) 36092762 PMC9449990

[39] EFSA Panel on Animal Health and Welfare (AHAW) et al. 2022 Welfare of small ruminants during transport. EFSA J. **20**, e07404. (10.2903/j.efsa.2022.7404) 36092764 PMC9449987

[40] European Commission. 2021 Inception impact assessment: Ares(2020)6081753, pp. 6–9.

[41] Nielsen BL, Dybkjr L, Herskin MS. 2011 Road transport of farm animals: effects of journey duration on animal welfare. Animal **5**, 415-427. (10.1017/S1751731110001989) 22445408

[42] Grandin T. 2001 Perspectives on transportation issues: the importance of having physically fit cattle and pigs. J. Anim. Sci. **79**, E201. (10.2527/jas2001.79e-supple201x)

[43] Cockram MS. 2019 Fitness of animals for transport to slaughter. Can. Vet. J. **60**, 423-429. 30992599 PMC6417610

[44] Herskin MS, Gerritzen MA, Michael M, Bracke MBM, Spoolder HAM. 2021 Review of fitness for transport of pigs. European Union Reference Centre for Animal Welfare Pigs (EURCAW-Pigs) **23**, 1-21.

[45] Stojkov J, von Keyserlingk MAG, Duffield T, Fraser D. 2020 Management of cull dairy cows: culling decisions, duration of transport, and effect on cow condition. J. Dairy Sci. **103**, 2636-2649. (10.3168/jds.2019-17435) 31954571

[46] González LA, Schwartzkopf-Genswein KS, Bryan M, Silasi R, Brown F. 2012 Relationships between transport conditions and welfare outcomes during commercial long haul transport of cattle in North America. J. Anim. Sci. **90**, 3640-3651. (10.2527/jas.2011-4796) 22665659

[47] Thodberg K, Fogsgaard KK, Herskin MS. 2019 Transportation of cull sows—deterioration of clinical condition from departure and until arrival at the slaughter plant. Front. Vet. Sci. **6**, 28. (10.3389/fvets.2019.00028) 30834251 PMC6387918

[48] González LA, Schwartzkopf-Genswein KS, Bryan M, Silasi R, Brown F. 2012 Benchmarking study of industry practices during commercial long haul transport of cattle in Alberta, Canada. J. Anim. Sci. **90**, 3606-3617. (10.2527/jas.2011-4770) 22665628

[49] Animal Welfare Institute. 2020 *Legal protections for farm animals during transport*.

[50] Hendricks J, Roche S, Proudfoot KL, von Keyserlingk MAG. 2023 Livestock haulers' views about dairy cattle transport in Atlantic Canada. J. Dairy Sci. **106**, 3548-3558. (10.3168/jds.2022-22752) 37002134

[51] Kuo C, von Keyserlingk MA. 2023 Livestock hauler and dairy farmer perspectives about cull dairy cattle transport and cattle transport regulations in British Columbia, Canada. Anim. Welf. **32**, e42. (10.1017/awf.2023.36) PMC10936252 38487451

[52] Marshall J, Haley D, Levison L, Kelton DF, Miltenburg C, Roche S, Duffield TF. 2022 A survey of dairy cattle farmers’ management practices for cull cows in Ontario, Canada. Front. Vet. Sci. **9**, 974061. (10.3389/fvets.2022.974061) 36110502 PMC9468542

[53] Marshall J, Haley DB, Kelton D, Miltenburg C, Roche S, Duffield T. 2023 A focus group study exploring dairy farmers' perspectives of cull cow management in Ontario, Canada. Front. Vet. Sci. **10**, 1189668. (10.3389/fvets.2023.1189668) 37346277 PMC10279770

[54] Marshall J, Haley D, Levison L, Kelton DF, Miltenburg C, Roche S, Duffield TF. 2022 A survey of practices and attitudes around cull cow management by bovine veterinarians in Ontario, Canada. J. Dairy Sci. **106**, 302-311. (10.3168/jds.2022-22005) 36333137

[55] Grandin T. 2016 Transport fitness of cull sows and boars: a comparison of different guidelines on fitness for transport. Animals **6**, 77. (10.3390/ani6120077) 27916798 PMC5187500

[56] Dahl-Pedersen K. 2022 Danish cattle farmers’ experience with fitness for transport—a questionnaire survey. Front. Vet. Sci. **9**, 797149. (10.3389/fvets.2022.797149) 35372551 PMC8971744

[57] Herskin MS, Hels A, Anneberg I, Thomsen PT. 2017 Livestock drivers' knowledge about dairy cow fitness for transport—a Danish questionnaire survey. Res. Vet. Sci. **113**, 62-66. (10.1016/j.rvsc.2017.09.008) 28892662

[58] Dahl-Pedersen K, Foldager L, Herskin MS, Houe H, Thomsen PT. 2018 Lameness scoring and assessment of fitness for transport in dairy cows: agreement among and between farmers, veterinarians and livestock drivers. Res. Vet. Sci. **119**, 162-166. (10.1016/j.rvsc.2018.06.017) 29940460

[59] European Commission, Food Safety. 2015 Overview report systems to prevent the transport of unfit animals in the EU. (10.2875/45032)

[60] Werner C, Reiners K, Wicke M. 2007 Short as well as long transport duration can affect the welfare of slaughter pigs. Anim. Welf. **16**, 385-389. (10.1017/S0962728600027202)

[61] Malena M, Voslářová E, Tomanová P, Lepková R, Bedáñová I, Večerek V. 2006 Influence of travel distance and the season upon transport-induced mortality in fattened cattle. Acta Vet. Brno **75**, 619-624. (10.2754/avb200675040619)

[62] Simova V, Voslarova E, Vecerek V, Passantino A, Bedanova I. 2017 Effects of travel distance and season of the year on transport-related mortality in cattle. Anim. Sci. J. **88**, 526-532. (10.1111/asj.12658) 27460957

[63] Cave JG, Callinan APL, Woonton WK. 2005 Mortalities in bobby calves associated with long distance transport. Aust. Vet. J. **83**, 82-84. (10.1111/j.1751-0813.2005.tb12203.x) 15971826

[64] Vecerek V, Malena M, Malena M, Voslarova E, Chloupek P. 2006 The impact of the transport distance and season on losses of fattened pigs during transport to the slaughterhouse in the Czech Republic in the period from 1997 to 2004. Vet. Med. **51**, 21-28. (10.17221/5513-VETMED)

[65] Dewey CE, Haley C, Widowski T, Poljak Z, Friendship RM. 2009 Factors associated with in-transit losses of fattening pigs. Anim. Welf. **18**, 355-361. (10.1017/S0962728600000750) PMC2568040 19086368

[66] Rioja-Lang FC, Brown JA, Brockhoff EJ, Faucitano L. 2019 A review of swine transportation research on priority welfare issues: a Canadian perspective. Front. Vet. Sci. **6**, 36. (10.3389/fvets.2019.00036) 30854374 PMC6395376

[67] Sommavilla R et al. 2017 Season, transport duration and trailer compartment effects on blood stress indicators in pigs: relationship to environmental, behavioral and other physiological factors, and pork quality traits. Animals **7**, 8. (10.3390/ani7020008) 28208689 PMC5332929

[68] Roy RC, Cockram MS, Dohoo IR. 2015 Welfare of horses transported to slaughter in Canada: assessment of welfare and journey risk factors affecting welfare. Can. J. Anim. Sci. **95**, 509-522. (10.4141/cjas-2015-031)

[69] Chulayo AY, Bradley G, Muchenje V. 2016 Effects of transport distance, lairage time and stunning efficiency on cortisol, glucose, HSPA1A and how they relate with meat quality in cattle. Meat Sci. **117**, 89-96. (10.1016/j.meatsci.2016.03.001) 26967002

[70] Friend TH. 2000 Dehydration, stress, and water consumption of horses during long-distance commercial transport. J. Anim. Sci. **78**, 2568-2580. (10.2527/2000.78102568x) 11048922

[71] Stojkov J, Bowers G, Draper M, Duffield T, Duivenvoorden P, Groleau M, Haupstein D. 2018 Hot topic: management of cull dairy cows—consensus of an expert consultation in Canada. J. Dairy Sci. **101**, 11 170-11 174. (10.3168/jds.2018-14919) 30243623

[72] van Dyke R, Connor M, Miele A. 2021 An investigation into the perceptions of veterinarians towards perioperative pain management in calves. Animals **11**, 1-17. (10.3390/ani11071882) PMC8300249 34202730

[73] Bolton SE, von Keyserlingk MAG. 2021 The dispensable surplus dairy calf: is this issue a ‘wicked problem’ and where do we go from here? Front. Vet. Sci. **8**, 660934. (10.3389/fvets.2021.660934) 33937380 PMC8079806

[74] Roadknight N, Mansell P, Jongman E, Courtman N, Fisher A. 2021 Invited review: the welfare of young calves transported by road. J. Dairy Sci. **104**, 6343-6357. (10.3168/jds.2020-19346) 33714583

[75] Goetz HM, Creutzinger KC, Kelton DF, Costa JHC, Winder CB, Renaud DL. 2023 A randomized controlled trial investigating the effect of transport duration and age at transport on surplus dairy calves: Part I. Impact on health and growth. J. Dairy Sci. **106**, 2784-2799. (10.3168/jds.2022-22366) 36797186

[76] Appleby MC. 2008 Long distance transport and welfare of farm animals. Wallingford, UK: CABI.

[77] Driessen B, Freson L, Buyse J. 2020 Fasting finisher pigs before slaughter influences pork safety, pork quality and animal welfare. Animals **10**, 1-10. (10.3390/ani10122206) PMC7761097 33255610

[78] Fisher AD, Colditz IG, Lee C, Ferguson DM. 2009 The influence of land transport on animal welfare in extensive farming systems. J. Vet. Behav. Clin. Appl. Res. **4**, 157-162. (10.1016/j.jveb.2009.03.002)

[79] Cockram MS, Mitchell MA. 1999 Rôle of research in the formulation of ‘rules’ to protect the welfare of farm animals during road transportation. BSAP Occass. Publ. **23**, 43-64. (10.1017/S0263967X00033243)

[80] Cockram MS. 2007 Criteria and potential reasons for maximum journey times for farm animals destined for slaughter. Appl. Anim. Behav. Sci. **106**, 234-243. (10.1016/j.applanim.2007.01.006)

[81] Marti S, Wilde RE, Moya D, Heuston CEM, Brown F, Schwartzkopf-Genswein KS. 2017 Effect of rest stop duration during long-distance transport on welfare indicators in recently weaned beef calves. J. Anim. Sci. **95**, 636-644. (10.2527/jas.2016.0739) 28380612

[82] Meléndez DM, Marti S, Haley DB, Schwinghamer TD, Schwartzkopf-Genswein KS. 2020 Effect of transport and rest stop duration on the welfare of conditioned cattle transported by road. PLoS ONE **15**, e0228492. (10.1371/journal.pone.0228492) 32120382 PMC7051828

[83] Meléndez DM, Marti S, Haley DB, Schwinghamer TD, Schwartzkopf-Genswein KS. 2021 Effects of conditioning, source, and rest on indicators of stress in beef cattle transported by road. PLoS ONE **16**, e0244854. (10.1371/journal.pone.0244854) 33434915 PMC7803389

[84] Knowles G, Warriss PD, Brown SN, Edwards JE. 1999 Effects on cattle of transportation by road for up to 31 hours. Vet. Rec. **145**, 575-582. (10.1136/vr.145.20.575) 10606018

[85] Earley B, Drennan M, O'Riordan EG. 2013 The effect of road transport in comparison to a novel environment on the physiological, metabolic and behavioural responses of bulls. Res. Vet. Sci. **95**, 811-818. (10.1016/j.rvsc.2013.04.027) 23726664

[86] Messori S, Pedernera-Romano C, Magnani D, Rodriguez P, Barnard S, Dalmau A, Velarde A, Dalla Villa P. 2015 Unloading or not unloading? Sheep welfare implication of rest stop at control post after a 29 h transport. Small Ruminant Res. **130**, 221-228. (10.1016/j.smallrumres.2015.07.012)

[87] Cockram MS et al. 2000 Behavioural and physiological responses of sheep to 16 h transport and a novel environment post-transport. Vet. J. **159**, 139-146. (10.1053/tvjl.1999.0411) 10712801

[88] Broom DM. 2008 The welfare of livestock during road transport. In Long distance transport and welfare of farm animals, pp. 157-181. Wallingford, UK: CABI.

[89] Parrott R, Hall S, Lloyd D, Goode J, Broom D. 1998 Effects of a maximum permissible journey time (31 h) on physiological responses of fleeced and shorn sheep to transport, with observations on behaviour during a short (1 h) rest-stop. Anim. Sci. **66**, 197-207. (10.1017/S1357729800008961)

[90] Cockram MS et al. 1997 Effect of lairage during 24 h of transport on the behavioural and physiological responses of sheep. Anim. Sci. **65**, 391-402. (10.1017/S1357729800008596)

[91] Messori S, Pedernera-Romano C, Rodriguez P, Barnard S, Giansante D, Magnani D, Dalmau A, Velarde A, Dalla Villa P. 2017 Effetto dei diversi tempi di sosta al posto di controllo durante il lungo viaggio sul benessere delle pecore. Vet. Ital. **53**, 121-129. (10.12834/VetIt.316.1483.3) 28675250

[92] Doyle R, Roest HJ, Sultana P, Tullio D, Baumgärtner I, Velarde A. 2021 Public hearing on long distance transports of live animals within the European Union.

[93] Duthoit S. 2017 Le transport « longue durée » de bovins vivants: les incohérences de la règlementation européenne régulièrement pointées du doigt par la Cour de justice de l'Union européenne. Европейски правен преглед. See https://evropeiskipravenpregled.eu/t188/ (accessed 8 February 2023).

[94] Thodberg K, Foldager L, Fogsgaard KK, Gaillard C, Herskin MS. 2022 Temperature conditions during commercial transportation of cull sows to slaughter. Comp. Electron. Agric. **192**, 106626. (10.1016/j.compag.2021.106626)

[95] Goldhawk C, Janzen C, González LA, Crowe T, Kastelic J, Kehler C, Siemens M, Schwartzkopf-Genswein FM, Pajor FM. 2015 Trailer temperature and humidity during winter transport of cattle in Canada and evaluation of indicators used to assess the welfare of cull beef cows before and after transport. J. Anim. Sci. **93**, 3639-3653. (10.2527/jas.2014-8390) 26440030

[96] Fisher AD, Stewart M, Duganzich DM, Tacon J, Matthews LR. 2004 The effects of stationary periods and external temperature and humidity on thermal stress conditions within sheep transport vehicles. N Z Vet. J. **53**, 6-9. (10.1080/00480169.2005.36461) 15731827

[97] Knezacek TD, Olkowski AA, Kettlewell PJ, Mitchell MA, Classen HL. 2010 Temperature gradients in trailers and changes in broiler rectal and core body temperature during winter transportation in Saskatchewan. Can. J. Anim. Sci. **90**, 321-330. (10.4141/cjas09083)

[98] Schwartzkopf-Genswein K, Ahola J, Edwards-Callaway L, Hale D, Paterson J. 2016 Transportation issues affecting cattle well-being and considerations for the future. Prof. Anim. Sci. **32**, 707-716. (10.15232/pas.2016-01517)

[99] De la Fuente J, Díaz MT, Ibáñez M, González de Chavarri E. 2007 Physiological response of rabbits to heat, cold, noise and mixing in the context of transport. Anim. Welf. **16**, 41-47. (10.1017/S0962728600030918)

[100] Mitchell MA, Kettlewell PJ. 2009 Welfare of poultry during transport—a review. In *Poultry Welfare Symp., Cervia, Italy, 18–22 May 2009*.

[101] Nielsen SS et al. 2022 Methodological guidance for the development of animal welfare mandates in the context of the Farm to Fork Strategy. EFSA J. **20**, e07403. (10.2903/j.efsa.2022.7403) 35846109 PMC9275173

[102] Caulfield MP, Cambridge H, Foster SF, McGreevy PD. 2014 Heat stress: a major contributor to poor animal welfare associated with long-haul live export voyages. Vet. J. **199**, 223-228. (10.1016/j.tvjl.2013.09.018) 24157340

[103] Purwanto BP, Abo Y, Sakamoto R, Yamamoto S, Furumoto F. 1990 Diurnal patterns of heat production and heart rate under thermoneutral conditions in Holstein Friesian cows differing in milk production. J. Agric. Sci. **114**, 139-142. (10.1017/S0021859600072117)

[104] Mitchell MA, Kettlewell PJ. 2008 Engineering and design of vehicles for long distance road transport of livestock (ruminants, pigs and poultry). Vet. Ital. **44**, 201-213. 20405426

[105] Sutherland MA, McDonald A, McGlone JJ. 2009 Effects of variations in the environment, length of journey and type of trailer on the mortality and morbidity of pigs being transported to slaughter. Vet. Rec. **165**, 13-18. (10.1136/vetrec.165.1.13) 19578189

[106] Haley C, Dewey CE, Widowski T, Poljak Z, Friendship R. 2008 Factors associated with in-transit losses of market hogs in Ontario in 2001. Can. J. Vet. Res. **72**, 377-384. 19086368 PMC2568040

[107] Gade PB, Christensen L, Baltzer M, Petersen JV. 2007 Causes of pre-slaughter mortality in Danish slaughter pigs with special emphasis on transport. Anim. Welf. **16**, 459-470. (10.1017/S0962728600027391)

[108] Warriss PD. 1998 The welfare of slaughter pigs during transport. Anim. Welf. **7**, 365-381. (10.1017/S0962728600020923)

[109] Robbins LA, Green-Miller AR, Lay DC, Schinckel AP, Johnson JS, Gaskill BN. 2021 Evaluation of sow thermal preference across three stages of reproduction. J. Anim. Sci. **99**, 1-10. (10.1093/jas/skab202) PMC8355610 34197578

[110] Bjerg B, Brandt P, Pedersen P, Zhang G. 2020 Sows' responses to increased heat load—a review. J. Therm. Biol **94**, 102758. (10.1016/j.jtherbio.2020.102758) 33292999

[111] Bracke MBM, Herskin MS, Marahrens M, Gerritzen MA, Spoodler HAM. 2020 Review of climate control and space allowance during transport of pigs (version 1.0). Wageningen, The Netherlands: EURCAW Pigs.

[112] US Department of Commerce. In press. What is the heat index? See https://www.weather.gov/ama/heatindex (accessed 4 July 2023).

[113] Habeeb AA, Gad AE, Atta MA. 2018 Temperature-humidity indices as indicators to heat stress of climatic conditions with relation to production and reproduction of farm animals. Int. J. Biotechnol. Rec. Adv. **1**, 35-50. (10.18689/ijbr-1000107)

[114] Collins T, Hampton JO, Barnes AL. 2018 A systematic review of heat load in Australian livestock transported by sea. Animals **8**, 1-16. (10.3390/ani8100164) PMC6210166 30261695

[115] Carnovale F, Phillips CJC. 2020 The effects of heat stress on sheep welfare during live export voyages from Australia to the Middle East. Animals **10**, 694. (10.3390/ani10040694) 32316242 PMC7222853

[116] McCarthy M. 2018 *Independent review of conditions for the export of sheep to the Middle East during the northern hemisphere summer*. Canberra, Australia: Department of Agriculture and Water Resources.

[117] Ellis E. 2022 Australian animal law: context and critique. Sydney, Australia: Sydney University Press.

[118] Australian Government. 2022 *Review of live sheep exports by sea to, or through, the Middle East during the Northern Hemisphere summer*. Canberra, Australia: Department of Agriculture, Fisheries and Forestry.

[119] Miranda-de la Lama GC, Villarroel M, María GA. 2014 Livestock transport from the perspective of the pre-slaughter logistic chain: a review. Meat Sci. **98**, 9-20. (10.1016/j.meatsci.2014.04.005) 24824530

[120] Warris PD, Brown SN, Knowles TG, Wilkins LJ, Pope SJ, Chadd SA, Kettlewell PJ, Green NR. 2006 Comparison of the effects of fan-assisted and natural ventilation of vehicles on the welfare of pigs being transported to slaughter. Vet. Rec. **158**, 585-588. (10.1136/vr.158.17.585) 16648438

[121] Schwartzkopf-Genswein K, Grandin T. 2014 Cattle transport by road. In Livestock handling and transport (ed. T Grandin), pp. 143-173. Wallingford, UK: CABI.

[122] Scientific Committee on Animal Health and Animal Welfare. 2002 The welfare of animals during transport (details for horses, pigs, sheep and cattle). Anim. Welf. **11**, 354-355. (10.1017/S0962728600024982)

[123] Tarrant PV, Kenny FJ, Harrington D. 1988 The effect of stocking density during 4 hour transport to slaughter on behaviour, blood constituents and carcass bruising in Friesian steers. Meat Sci. **24**, 209-222. (10.1016/0309-1740(88)90079-4) 22055952

[124] Tarrant PV, Kenny FJ, Harrington D, Murphy M. 1992 Long distance transportation of steers to slaughter: effect of stocking density on physiology, behaviour and carcass quality. Livest. Prod. Sci. **30**, 223-238. (10.1016/S0301-6226(06)80012-6)

[125] Visser K. 2014 Note on minimum space allowance and compartment height for cattle and pigs during transport. Wageningen, The Netherlands: Livestock Research.

[126] Consortium of the Animal Transport Guides. 2018 *Guide to good practices for the transport of cattle*.

[127] EFSA Panel on Animal Health and Welfare (AHAW). 2011 Scientific opinion concerning the welfare of animals during transport. EFSA J. **9**, 1966. (10.2903/j.efsa.2011.1966)

[128] FAWC. 2019 The welfare of animals during transport. Gov 1-30.

[129] Petherick JC, Phillips CJC. 2009 Space allowances for confined livestock and their determination from allometric principles. Appl. Anim. Behav. Sci. **117**, 1-12. (10.1016/j.applanim.2008.09.008)

[130] Knowles TG, Brown SN, Pope SJ, Nicol CJ, Warriss PD, Weeks CA. 2010 The response of untamed (unbroken) ponies to conditions of road transport. Anim. Welf. **19**, 1-15. (10.1017/S096272860000110X)

[131] Warriss PD, Brown SN, Knowles TG, Kestin SC, Edwards JE, Dolan SK, Phillips AJ. 1995 Effects on cattle of transport by road for up to 15 h. Vet. Rec. **136**, 319-323. (10.1136/vr.136.13.319) 7604507

[132] Visser. 2014 Behaviour of heifers during long distance transport behaviour of heifers during long distance transport.

[133] Animals’ Angels. 2021 100 reasons to revise Council Regulation EC 1/2005 on the protection of animals during transport.

[134] Menchetti L, Costa LN, Zappaterra M, Padalino B. 2021 Effects of reduced space allowance and heat stress on behavior and eye temperature in unweaned lambs: a pilot study. Animals **11**, 1-19. (10.3390/ani11123464) PMC8698074 34944241

[135] Arndt H, Volkmann N, Spindler B, Hartung J, Kemper N. 2019 Do pigs have adequate space in animal transportation vehicles? Planimetric measurement of the floor area covered by finishing pigs in various body positions. Front. Vet. Sci. **5**, 330. (10.3389/fvets.2018.00330) 30687722 PMC6335254

[136] FAWC. 2013 FAWC advice on space and headroom allowances for transport of farm animals.

[137] European Commission. 2023/0448 Proposal for a Regulation of the European Parliament and of the Council on the protection of animals during transport and related operations, amending Council Regulation (EC) No 1255/97 and repealing Council Regulation (EC) No 1/2005.

[138] European Parliament. Directorate General for Parliamentary Research Services. 2018 Regulation (EC) No 1/2005 on the protection of animals during transport and related operations: European implementation assessment. Strasbourg, France: EU Publications Office.

[139] Wilhelmsson S. 2022 *There's no time to rush!: pigs' and transport drivers’ welfare and interactions during slaughter transport*.

[140] Defra WG. 2021 Improvements to animal welfare in transport: summary of responses and government response.

[141] Court of Justice of the European Union C-424/13 2015 Zuchtvieh-Export GmbH v Stadt Kempten.

[142] Animal Welfare (Livestock Exports) Bill 2023.

[143] Department for Environment, Food and Rural Affairs. 2021 Live animal exports, impact assessment. See https://publications.parliament.uk/pa/bills/cbill/58-02/0013/LiveAnimalExportFinalStageImpactAssessment.pdf (accessed 14 December 2023).

[144] 2022 Tiertransporte aus Deutschland werden deutlich eingeschränkt. *BMEL*. See https://www.bmel.de/SharedDocs/Pressemitteilungen/DE/2022/148-tiertransporte.html (accessed 12 February 2023).

[145] Council of the European Union, General Secretariat of the Council. 2022 Updating legislation on the transport of animals in the EU. Information from the Belgian, Danish, Dutch, German and Swedish delegations.

[146] 2022 Live sheep trade ban won't happen in this term, PM says. *ABC News*, 3 June. See https://www.abc.net.au/news/2022-06-03/sheep-live-export-ban-labor-agriculture-minister-confirms/101119752.

[147] Offor I. 2020 Animals and the impact of trade law and policy: a global animal law question. Transnat. Environ. Law **9**, 239-262. (10.1017/S2047102519000402)

[148] Consortium of the Animal Transport Guides. 2018 Guide to good practices for the transport of sheep.

[149] Shroff V. 2021 Canadian animal law book. New York, NY: LexisNexis.

[150] Rodriguez Ferrere M, King M, Larsen LM. 2019 Animal welfare in New Zealand: Oversight, compliance and enforcement.

[151] Marlin D, Kettlewell P, Parkin T, Kennedy M, Broom D, Wood J. 2011 Welfare and health of horses transported for slaughter within the European Union Part 1: methodology and descriptive data. Equine Vet. J. **43**, 78-87. (10.1111/j.2042-3306.2010.00124.x) 21143638

[152] Padalino B, Menchetti L, Mininni V, Tullio D, Nanni Costa L. 2021 Transport certifications of cattle moved from France to Southern Italy and Greece: do they comply with Reg. EC 1/2005? Ital. J. Anim. Sci. **20**, 1870-1881. (10.1080/1828051X.2021.1971573)

[153] Schwartzkopf-Genswein K, Grandin T. 2019 Cattle transport in North America. In Livestock handling and transport (ed. T Grandin), pp. 153-183. Wallingford, UK: CABI.

[154] Anneberg I, Sandøe P. 2019 When the working environment is bad, you take it out on the animals—how employees on Danish farms perceive animal welfare. Food Ethics **4**, 21-34. (10.1007/s41055-019-00044-6)

[155] Wilhelmsson S, Andersson M, Arvidsson I, Dahlqvist C, Hemsworth PH, Yngvesson J, Hultgren J. 2021 Physical workload and psychosocial working conditions in Swedish pig transport drivers. Int. J. Ind. Ergon. **83**, 103124. (10.1016/j.ergon.2021.103124)

[156] Federation of Veterinarians of Europe. 2016 FVE calls to end suffering of animals during long distance transports.

[157] Baltussen WHM, Spoolder HAM, Lambooij E, Backus GBC. 2009 Sustainable production: transporting animals or meat?

[158] Hultgren J. 2018 Is livestock transport a necessary practice? Mobile slaughter and on-farm stunning and killing before transport to slaughter. CABI Rev. **13**, 1-5. (10.1079/PAVSNNR201813054)

[159] Eriksen MS, Rødbotten R, Grøndahl AM, Friestad M, Andersen IL, Mejdell CM. 2013 Mobile abattoir versus conventional slaughterhouse—impact on stress parameters and meat quality characteristics in Norwegian lambs. Appl. Anim. Behav. Sci. **149**, 21-29. (10.1016/j.applanim.2013.09.007)

[160] Herskin MS, Duffield TF. 2020 Editorial: animal transport related management. J. Dairy Sci. **101**, 11 170-11 174. (10.3389/fvets.2020.614317) PMC7728605 33330728

[161] Creutzinger KC, Broadfoot K, Goetz HM, Proudfoot KL, Costa JHC, Meagher RK, Renaud DL. 2022 Assessing dairy calf response to long-distance transportation using conditioned place aversion. JDS Commun. **3**, 275-279. (10.3168/jdsc.2022-0209) 36338020 PMC9623717

[162] Birch J. 2017 Animal sentience and the precautionary principle. Anim. Sent. **2**, 1. (10.51291/2377-7478.1200)

[163] Duval E, Lecorps B, von Keyserlingk MAG. 2024 Are regulations addressing farm animal welfare issues during live transportation fit for purpose? A multi-country jurisdictional check. Figshare. (10.6084/m9.figshare.c.7007824) PMC10805601 38269076

